# A Rare Case of Four Primary Tumors in a Patient With Lynch Syndrome

**DOI:** 10.1002/ccr3.70560

**Published:** 2025-06-02

**Authors:** Mostafa Kamandi, Negin Layegh, Hamideh Feiz Disfani

**Affiliations:** ^1^ Department of Internal Medicine, Faculty of Medicine Mashhad University of Medical Sciences Mashhad Iran; ^2^ Innovative Medical Research Center, Faculty of Medicine, Mashhad Medical Sciences Islamic Azad University Mashhad Iran; ^3^ Department of Emergency Medicine, Faculty of Medicine Mashhad University of Medical Sciences Mashhad Iran

**Keywords:** case report, colon cancer, lymphoma, Lynch syndrome, multiple primary tumors

## Abstract

This case emphasizes the complexity of LS and the need for more comprehensive surveillance strategies, particularly in those with MPTs, to enable early detection and improve management.

## 1 Introduction

Multiple primary tumors (MPTs) are characterized by the presence of two or more synchronous or metachronous malignant tumors in the same individual [1]. The incidence of MPTs varies between 2.4% and 8%, up to 17% within 20 years of follow‐up [2]. Advances in cancer diagnosis and treatment have increased the likelihood of individuals developing MPTs as the number of cancer survivors continues to rise [3]. However, the occurrence of three or more tumors remains very rare [4]. Studies have shown that inherited predisposition to cancer, lifestyle, hormonal, and environmental factors are among the risk factors for the development of MPTs [5]. Lynch syndrome (LS) is the most common inherited disorder caused by germline mutations in DNA mismatch repair (MMR) genes: MLH1, MSH2, MSH6, and PMS2. The inactivation of both alleles in one of the MMR genes generates microsatellite instability (MSI), which increases the lifetime risk of several malignancies, most commonly colorectal and endometrial cancers [2], [6]. Here, we present a rare case of four metachronous malignancies in a patient with LS, including three solid tumors and one hematologic malignancy.

## 2 Case Presentation

### 2.1 Case History and Examination

A 39‐year‐old women with abdominal pain and vaginal bleeding presented to Mashhad University of Medical Sciences Department of Medical Oncology and Hematology in December 2019. Twenty years earlier, she had been diagnosed with B‐cell lymphoma and treated with the CHOP regimen (cyclophosphamide, doxorubicin hydrochloride, vincristine sulfate, and prednisone).

An abdominopelvic CT scan showed a heterogeneous mass (45 × 39 mm) in the left adnexa. Due to the patient's age and the desire to preserve fertility, a left salpingo‐oophorectomy was performed. Histopathology reported high‐grade papillary serous carcinoma without metastasis. The patient underwent a CT scan that identified a subserosal mass in the uterine fundus, suggestive of a uterine fibroid. The tumor markers carbohydrate antigen (CA) 19‐9 and CA125 were within normal ranges (28.2 and 12.5 U/mL, respectively). Chemotherapy, including six rounds of paclitaxel and carboplatin, was administered. The patient was followed up since and had no significant complications.

**FIGURE 1:**
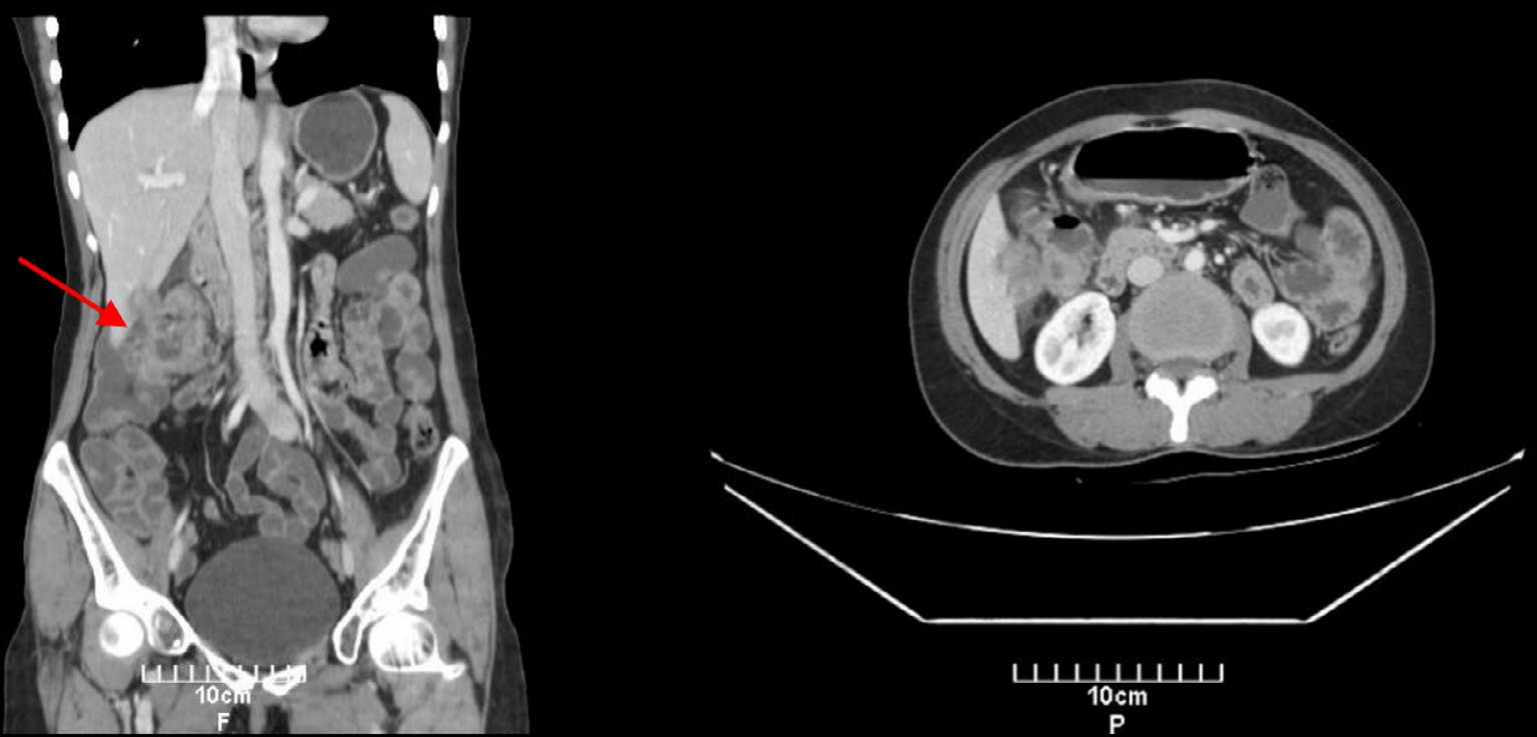
Contrast‐enhanced CT scan of the abdomen demonstrating a soft‐tissue mass in the ascending colon with circumferential wall thickening (arrow). There is local invasion to the liver and enlarged regional lymph nodes, suggestive of locally advanced colon cancer.

Four years later, she returned with abdominal pain. A CT scan of the abdomen showed a large tumoral mass in the ascending colon with circumferential wall thickening and luminal narrowing. Local invasion to the liver and multiple regional lymphadenopathies were present, suggestive of locally advanced colon cancer without evidence of distant metastasis (Figure 1).

### 2.2 Investigations and Treatment

Colonoscopy was performed, and a colon biopsy revealed moderately differentiated adenocarcinoma. The carcinoembryonic antigen (CEA) level was 1.5 ng/mL (normal range: 0–2.5 ng/mL). Following standard protocol, the patient was evaluated for LS. Immunohistochemistry (IHC) was performed for PMS2, MLH1, MSH2, and MSH6, revealing a loss of PMS2 expression. Fluorescence in situ hybridization (FISH) confirmed a PMS2 gene deletion, consistent with LS. Due to financial limitations, the patient could not receive immunotherapy. Neoadjuvant chemotherapy of the mFOLFOX6 regimen (folinic acid, fluorouracil, and oxaliplatin) was administered. The patient was then referred to the surgical department to undergo right hemicolectomy. Since she was diagnosed with LS, prophylactic hysterectomy and right salpingo‐oophorectomy were also performed.

### 2.3 Outcome and Follow‐Up

Postoperative pathological findings included foreign body reaction without viable cancer cells in the colon, and the International Federation of Gynecology and Obstetrics (FIGO) stage 1A endometrial endometrioid adenocarcinoma. There was less than 50% myometrial invasion without lymphovascular invasion; the ovary and fallopian tube were not involved. After the surgery, chemotherapy was completed. The patient is currently under follow‐up, and recent laboratory tests and CT scans report no cancer recurrence.

Table 1 provides an overview of the LS‐associated cancers diagnosed in this patient.

**TABLE 1:** Overview of primary malignancies.

Malignancy	Age at diagnosis	Location	Pathology	Invasion/metastasis	Treatment
B‐cell lymphoma	19	Nodal	B‐cell lymphoma	No distant spread	CHOP chemotherapy
Ovarian papillary serous carcinoma	39	Left adnexa	High‐grade papillary serous	No metastasis	Left salpingo‐oophorectomy + carboplatin/paclitaxel
Colorectal adenocarcinoma	43	Ascending colon	Moderately differentiated adenocarcinoma	Local liver invasion; regional lymph nodes	mFOLFOX6+right hemicolectomy
Endometrial endometrioid adenocarcinoma	43	Uterine fundus	Endometrioid adenocarcinoma	< 50% myometrial invasion, no lymphovascular invasion	Prophylactic hysterectomy + right salpingo‐oophorectomy

## 3 Discussion and Conclusion

LS, previously known as hereditary non‐polyposis colorectal cancer (HNPCC), is an autosomal dominant disorder caused by mutations in MMR genes [7]. LS is associated with an increased risk of colorectal and endometrial cancers and, more rarely, cancers of the urinary tract, ovary, stomach, and small intestine [8]. Studies have suggested that inherited predisposition syndromes such as LS elevate the risk of MPTs. However, the presentation of four or more MPTs is very rare [4, 9].

LS is due to germline mutations in MLH1, MSH2, MSH6, and PMS2. These genes are involved in the DNA mismatch repair system [10]. These mutations cause an inability to correct replication errors, particularly in microsatellite regions. This condition, known as microsatellite instability (MSI), is the hallmark of LS [11]. MSI leads to increased mutations in tumor suppressor genes and oncogenes, further promoting cancer development [12]. In this patient, we found loss of PMS2 expression through IHC and FISH, consistent with LS. While PMS2 mutations are associated with milder cancer risks compared with other MMR gene mutations, their role in early‐onset and more aggressive malignancies highlights the importance of genetic testing and surveillance [13].

There are well‐established screening guidelines for classic LS‐associated cancers, but recommendations for less common tumors remain limited. For colorectal cancer, colonoscopy begins every 1–2 years from the age of 20 to 25, and for endometrial cancer, transvaginal ultrasound and annual endometrial biopsy have been suggested [14]. In cases with a family history of gastric cancer or those of Asian descent, upper endoscopy may be considered every 1–3 years. Capsule endoscopy and small bowel enterography may be beneficial to symptomatic patients, and annual urinalysis may be considered for urothelial cancer surveillance [10]. This case emphasizes the need for broader surveillance strategies to improve early detection and management of all LS‐associated cancers.

The diagnosis of LS in this patient was not established until the evaluation of her fourth primary tumor. Earlier diagnoses of lymphoma and ovarian cancer did not prompt genetic testing, likely due to the atypical presentation, the rarity of hematologic malignancies in LS, and financial limitations. Standard screening protocols (IHC for MMR proteins in all colorectal and endometrial tumors) and emerging liquid biopsy approaches to obtain DNA‐ and RNA‐based biomarkers can facilitate the diagnosis of both classical and atypical LS‐associated cancers [15].

One interesting finding during the management of this case was the unexpected discovery of endometrial cancer during surgery. Most guidelines recommend prophylactic hysterectomy with bilateral salpingo‐oophorectomy as a viable option to prevent endometrial and ovarian cancers in women with LS [16]. However, more research is needed to confirm the effectiveness of this strategy.

Hematologic malignancies are rarely presented in patients with LS, with only a few documented cases. Studies suggest mutations in MMR genes, especially MSH6 deficiency, may contribute to lymphoma development and less typical tumor presentations [17]. Zhu et al. reported a case of LS with acute non‐lymphocytic leukemia, investigating the possible link between MMR defects and hematologic malignancies, particularly in conditions such as constitutional mismatch repair deficiency syndrome (CMMRD) [18, 19]. Further research is required to establish the exact underlying mechanisms.

This case demonstrates a rare presentation of four metachronous primary tumors in a patient with LS. While LS is well‐known as a predisposition to colorectal and endometrial cancers, the occurrence of MPTs, including hematologic malignancies, remains poorly understood. This case highlights the complexity of LS and the need for more tailored surveillance protocols to improve early detection and management in individuals with LS.

## Author Contributions

**Mostafa Kamandi:** data curation, project administration, writing – review and editing. **Negin Layegh:** data curation, investigation, writing – original draft, writing – review and editing. **Hamideh Feiz Disfani:** investigation, writing – original draft.

## Consent

The patient's written consent for the publication of this case report was obtained.

## Conflicts of Interest

The authors declare no conflicts of interest.

## Data Availability

The data that support the findings of this study are available from the corresponding author upon reasonable request.
